# Genome-wide analysis of microsatellite and sex-linked marker identification in *Gleditsia sinensis*

**DOI:** 10.1186/s12870-020-02551-9

**Published:** 2020-07-17

**Authors:** Jianjun Li, Chenglin Ye

**Affiliations:** College of Life Science, Henan Normal University, Green Medicine Biotechnology Henan Engineering Laboratory, Engineering Technology Research Center of Nursing and Utilization of Genuine Chinese Crude Drugs in Henan Province, Xinxiang, 453007 China

**Keywords:** SSR, *Gleditsia sinensis* lam., Sex identification

## Abstract

**Background:**

*Gleditsia sinensis* Lam. (Leguminosae), a dioecious perennial arbor, demonstrates important medicinal properties and economic value. These properties can be harnessed depending on the sex of the plant. However, the sex of the plants is difficult to identify accurately through morphological methods before the flowering.

**Results:**

We used bulked segregant analysis to screen sex-specific simple sequence repeat (SSR) markers in *G. sinensis*. Five male and five female plants were pooled to form the male and female bulks, respectively, and subjected to whole-genome sequencing. After high-throughput sequencing, 5,350,359 sequences were obtained, in which 2,065,210 SSRs were searched. Among them, the number of duplicated SSRs was the highest. The male plants could reach 857,874, which accounted for 60.86% of the total number of male plants. The female plants could reach 1,447,603, which accounted for 56.25% of the total model of the female plants. Among all the nucleotide repeat types, the A/T-rich motif was the most abundant. A total of 309,516 female strain-specific SSRs were selected by clustering. After designing the primers, the male and female gene pools were amplified, and five pairs of primers (i.e., 27, 34, 36, 39, and 41) were found to amplify the differential bands in the male and female gene pools. Using the five pairs of primers, we performed PCR verification on 10 individuals of known sex, which constructed the gene pool. The female plants amplified a single fragment of lengths (i.e., 186, 305, 266, 203, and 260 bp) and no male plant strip, thereby completing the identification of the male and female sexes of the *G. sinensis.*

**Conclusions:**

This study provides accurate sex identification strategies between female and male plants, thus improving the utilization rate of *G. sinensis* resources.

## Background

*Gleditsia sinensis* Lam. is an important native tree species in China [1]. It can be used for environmental protection, landscaping, and as an economic tree species and wood. In addition, the *G. sinensis* has a high medicinal value, including antidiabetic, antihyperglycemic, antioxidant, anti-inflammatory, anticancer, and anticoagulant activities. *Gleditsiae Sinensis* Fructus, *Fructus Gleditsiae*, and *Abnormalis Gleditsiae* Spina are recorded in the Chinese Pharmacopoeia as medicinal parts of *G. sinensis*. The flavonoids and steroids of *Gleditsiae* Spina play an important role in tumor treatment [2, 3]. In addition to its anticancer and antiviral effects, saponins can decontaminate and foam and have been extensively used in manufacturing cosmetics and detergents, with high economic value.

With the deepening of research, market demand for *G. sinensis* has increased over the years. *G. sinensis* is a dioecious plant species. The female tree has a strong pod-forming ability and long vegetative period, whereas the male plant does not form pods. Therefore, an ideal ratio of male-to-female individuals must be maintained in producing *G. sinensis* to improve economic efficiency. Numerous male trees will be a waste of land and labor given their slow growth rate. However, the *G. sinensis* tree has a long juvenile period and typically takes 6–8 years to blossom and bear fruit. Thus, the sexuality of trees is difficult to identify in the vegetative stage. Although the sexuality based on the differences of their external characteristics, such as flower morphology and physiological metabolites, is easy to identify at the mature stage, the accuracy and reliability are not high. Therefore, developing sex-specific molecular markers is crucial for identifying male trees at the seedling stage before transplanting.

Simple sequence repeat (SSR), also called microsatellite, is a class of repetitive DNA sequences, which are particularly abundant in plant genomes and have important influence on the function and evolution of genomes and chromosomes [4, 5]. It is also a DNA molecular marker technology based on a PCR technology. SSR is a group of nucleotides composed of several nucleotide (typically one to six) repeats, and the sequence on each side of the SSR is generally a relatively conservative single-copy sequence [6]. SSR markers have several advantages, such as abundant quantity, high information, and co-dominance, over other molecular markers. Each locus is determined by the sequence of designed primers. Therefore, a collaboration for developing primers is convenient for different laboratories [7]. With the rapid accumulation of genome sequences in many plant species, numerous SSR markers have been emerging in many plant species, such as rice [8], cotton [9], and oilseed rape [10]. These SSR markers have been extensively used in genetic map construction, gene mapping, fingerprint mapping, and marker-assisted breeding [11]. In a previous study, we have identified SSRs and have analyzed the distribution and frequency of various base repeat sequences in the *G. sinensis* genome [12]. In the present study, we identify the sex-specific SSR markers by combining bulked segregant analysis (BSA) and next-generation sequencing. The identified markers will be useful for early identification of sex and breeding in *G. sinensis*, thereby helping in maintaining an ideal ratio of male-to-female plants in production and in saving land and increasing pod yield.

## Results

### Number and distribution of SSR loci in *G. sinensis*

In 5,350,359 sequences from Male and female, 2,065,210 (38.6%) sequences contained SSRs, thus suggesting that the SSRs are highly enriched; a total of 461,166 sequences contained more than one SSR, and 582,826 SSRs contained composite forms. Moreover, 3,627,691 scaffolds assembled from the male genome of *G. sinensis*. The total length of the sequence was 1,375,655,435 bp, and the number of sequences containing SSR was 933,173, with a frequency of 25.72%. Furthermore, the total number of identified SSRs was 1,409,677. In terms of distribution, 1 SSR per 976 bp was set. A total of 3,713,022 scaffolds assembled from the female genome, and the total length of the sequence was 1,359,709,142 bp. The number of sequences containing SSR was 1,464,359, with a frequency of 39.43%, and the total number of identified SSRs was 2,573,292. In the female *G. sinensis* genome distribution, 1 SSR loci per 929 bp was set. The specific SSR search results are summarized in Table 1.

**Table 1:** SSR results statistics

Category	Male plant	Female plant
Total Number of Sequences Examined	3,627,691	3,713,022
Total Size of Examined Sequences (bp)	1,375,655,435	1,359,709,142
Total Number of Identified SSRs	1,409,677	2,573,292
Number of SSR Containing Sequence	933,173	1,464,359
Number of Sequences Containing More Than One SSR	330,488	665,965
Number of SSRs Present In Compound Formation	418,789	1,006,474

### Statistics and analysis of the SSR motif types

**Fig. 1:**
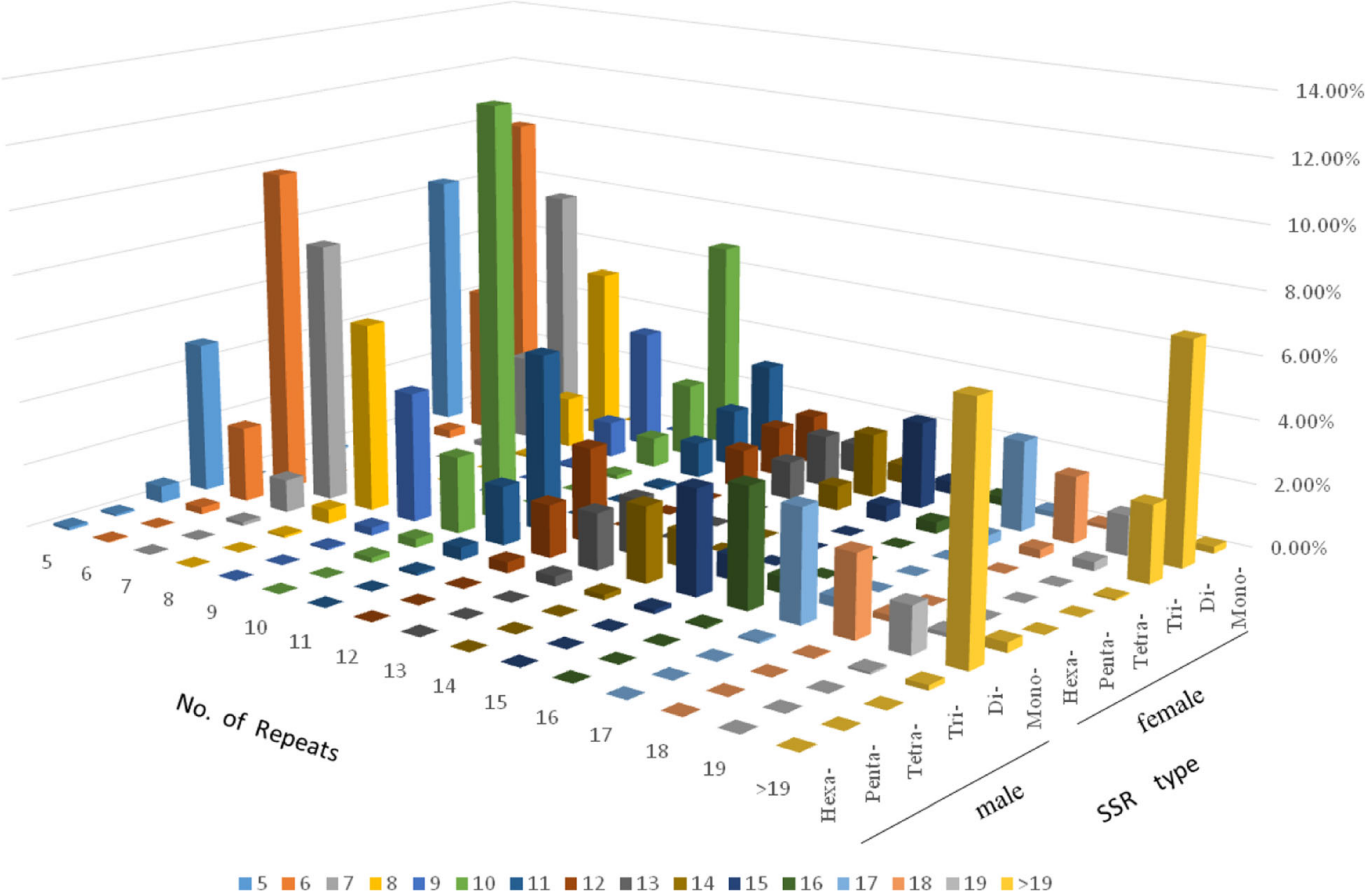
Distribution of SSR motif repeat numbers and relative frequency in the *G. sinensis* genome. The vertical axis shows the abundance of microsatellites with different motif repeat numbers (from 5 to > 19), which were discriminated by the legends of different colors

The different types of SSR motif were counted. The specific results are presented in Table 2. In this table, repeat SSRs from single-nucleotide repeat SSRs to six nucleotides were distributed in the male and female plants. Among them, the number of dinucleotide repeat motifs was the largest, with 857,874 males accounting for 60.86% of the total number of males and 1,447,603 females accounting for 56.25% of the total number of females. The number of mononucleotide and trinucleotide repeat motifs was also abundant, whereas the number of tetranucleotide to hexanucleotide repeat SSRs was small, and that of pentanucleotide repeat motifs was the least. There are 2200 pentanucleotide repeat motifs in female plants, accounting for 0.09% of the total SSR in female plants. The male plants had 1184 pentanucleotide repeat motifs, accounting for 0.08% of the total SSR of the male plants. Comparative analysis showed that genomic sequences of male and female plants are nearly distinct (Fig. 1). We further subcategorized each SSR repeating module in accordance with its sequence composition. The results of mononucleotide, dinucleotide, and trinucleotide repeat motifs are summarized in Table 3. In this table, the dominant repeat motifs of the mononucleotide repeat motifs in male and female plants are A/T. The dominant repetitive motif of male dinucleotide repeat motifs was AC/GT, female was AG/CT, and trinucleotide repeat motifs were mainly AAG/CTT.

**Table 2:** Percentage of SSR motifs

Type of SSR motif	Male plant		Female plant	
	SSR motif number	Percentage	SSR motif number	Percentage
Mononucleotide Repeat Motifs	377,120	26.75%	359,835	13.98%
Dinucleotide Repeat Motifs	857,874	60.86%	1,447,603	56.25%
Trinucleotide Repeat Motifs	153,104	10.86%	721,598	28.04%
Tetranucleotide Repeat Motifs	18,625	1.32%	38,623	1.50%
Pentanucleotide Repeat Motifs	1184	0.08%	2220	0.09%
Hexanucleotide Repeat Motifs	1770	0.13%	3413	0.13%
Total Repeat Motifs	1,409,677	100.00%	2,573,292	100.00%

**Table 3:** Results of Mononucleotide, Dinucleotide, and Trinucleotide Repeat Motifs

Repeat type	Male plant	Female plant
Mononucleotide	377,120	359,835
A/T	370,642	351,803
C/G	6478	8032
Dinucleotide	857,874	1,447,603
AC/GT	318,764	470,223
AG/CT	288,660	688,073
AT/AT	249,210	286,695
CG/CG	1240	2612
Trinucleotide	153,104	721,598
AAC/GTT	26,203	219,657
AAG/CTT	83,164	391,472
AAT/ATT	18,428	34,974
ACC/GGT	1665	6499
ACG/CGT	676	6892
ACT/AGT	606	3551
AGC/CTG	832	6492
AGG/CCT	18,014	36,867
ATC/ATG	3393	14,554
CCG/CGG	123	640

### SSR clustering and polymorphism analysis

The clustering results of SSRs are summarized in Table 4. 1,309,516 SSR sequences with a flanking length of more than 20 bp were selected from the sequence that contains SSRs, including 312,951 males and 516,961 females. The sequence of a specific SSR is clustered, and the result of clustering is displayed in Table 5.

**Table 4:** SSR clustering data

Items	Male plant		Female plant	
	Count	Percentage	Count	Percentage
SSR-Containing Sequences	933,173	100.00%	1,464,359	100.00%
SSR-Containing Sequences With Flanking Sequence Length ≥ 20 bp	312,951	33.54%	516,969	35.30%

**Table 5:** Statistics of female SSR clustering results

Items	Female specific SSR	
	Count	Percentage
SSR-Containing Sequence	241,081	100.00%
Cluster	122,999	51.02%

### SSR polymorphism assessment

In Table 6, a female-specific SSR length polymorphism was mainly concentrated in SSLP = 1, a total of 142,676, which account for 56.46% of the total. The contents of SSLP = 2 and SSLP = 3 were also high, accounting for 21.64 and 10.26%, respectively.

**Table 6:** SSR length polymorphism assessment

Items	Female specific SSR	
	Count of clusters	Percentage
SSLP = 1	142,676	56.46%
SSLP = 2	54,669	21.64%
SSLP = 3	25,913	10.26%
SSLP = 4	12,456	4.93%
SSLP = 5	5793	2.29%
SSLP = 6	2975	1.18%
SSLP = 7	1719	0.68%
SSLP = 8	1251	0.50%
SSLP = 9	842	0.33%
SSLP ≥10	4387	1.74%
Total	252,681	100.00%

*SSLP* Length polymorphism of SSR in the same class

### BSA pool PCR amplification results

We selected 50 pairs of random primers from the measured primers and amplified the PCR gene from the male and female gene pools. The amplified products were detected by 1% agarose gel electrophoresis. The results showed that 5 of the 50 pairs of primers (i.e., 27, 34, 36, 39, and 41) amplified different bands in the female gene pool but not in the male gene pool. The detailed information of the primers is presented in Table 7.

**Table 7:** Primer information and sequence

Primer	Upstream sequence	Downstream sequence	Tm (°C)	Size (bp)
Primer27	CGTCCGAGGACACGTAACTT	GCCGTAGAAGCAGAGCAGTT	60.17	186
Primer34	TCATCCACTGCGACTTTCAG	ACGTTTGCGTTGATACGTCA	59.98	305
Primer36	TTACCTAGATGGACCCGTGA	GGTTCTGTCCGTGCTGGTAT	58.02	266
Primer39	GCGTTACGCCACGTTATATG	AGGTTGGTCACAGGATACGC	59.13	203
Primer41	CTTGCGAGAGTCCAACATGA	CATGAAGGTTGACTCCGGTT	59.98	260

### PCR verification of known-sex plants

**Fig. 2:**
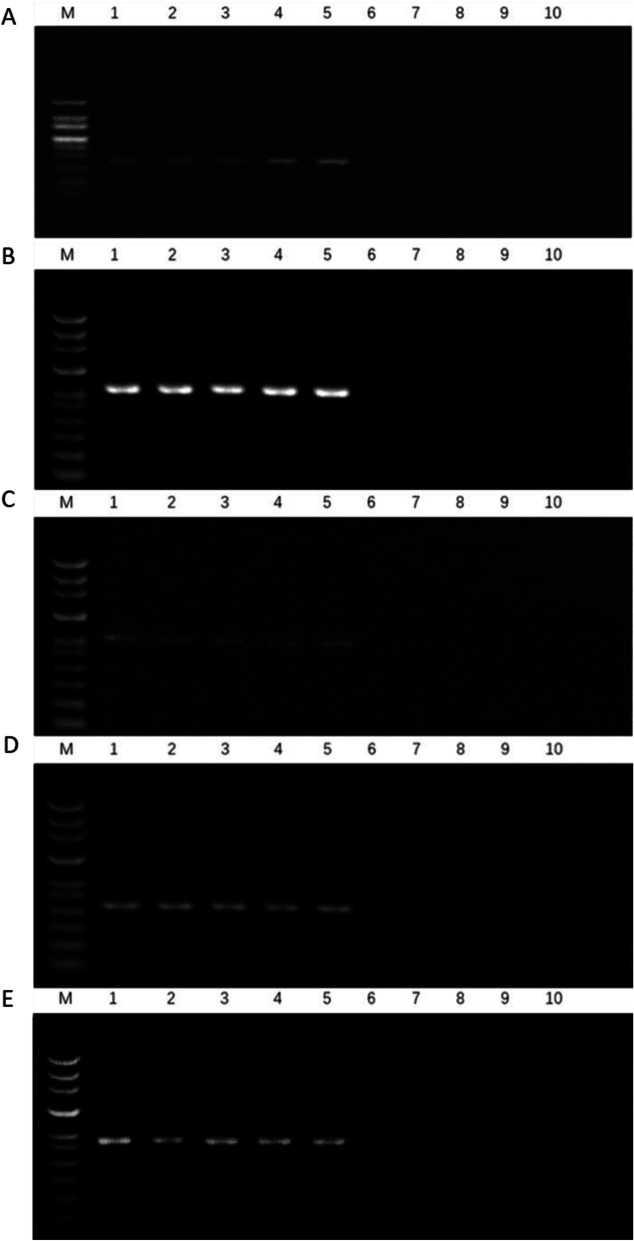
Amplification results of the five pairs of primers in 10 *G. sinensis*. **a** Primer27; **b** Primer34; **c** Primer36; **d** Primer39; **e** Primer41; (M) makerDL700; 1–5, female plant; 6–10, male plant

**Fig. 3:**
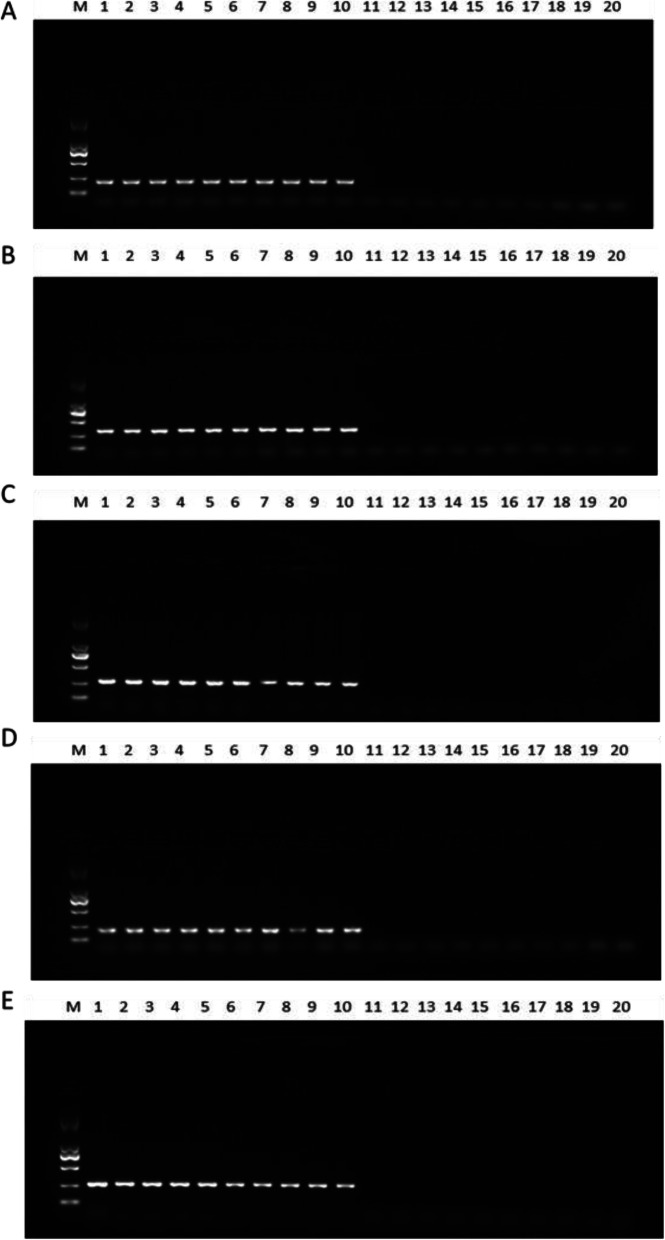
Amplification results of the five pairs of primers in 20 *G. sinensis*. **a** Primer27; **b** Primer34; **c** Primer36; **d** Primer39; **e** Primer41; (M) makerDL2000; 1–10, female plant; 11–20, male plant

Five pairs of screened primers were used to amplify PCR in 10 plants, which had previously constructed gene pools (Fig. 2). At the same time, five pairs of screened primers were used to amplify PCR in 20 plants from Xinxiang (Fig. 3). This comparison showed 100% accuracy in terms of sex identification. The female plants amplified a single fragment of lengths 186, 305, 266, 150, and 260 bp and no male plant strips.

## Discussion

With the rapid development of sequencing technology, numerous plant genomes are sequenced and published to the public database, thereby providing an excellent opportunity for searching SSR loci at the genome level and large-scale development of SSR markers. A bulk of previously published report demonstrates SSR markers based on genome sequencing data. For example, an average SSR locus per 1.14 kb is determined in *Arabidopsis thaliana* genome [13], and an average SSR locus per 4 kb is obtained in cabbage genome [14]. In the present study, 2,065,210 SSR loci are found in the genome of *G. sinensis*, with an average of 1 SSR locus per 976 bp for males and 1 SSR locus per 929 bp for females. Considering that only part of the gene sequence of *G. sinensis* is used, the results may not be directly compared with SSR data of other plants mentioned above. However, the results reveal that SSR is extensively distributed in the *G. sinensis* genome.

In the present study, differences in the types of SSR dominant motif are observed in most plants. Most of them are mainly dinucleotide and trinucleotide repeat motifs. Among them, dinucleotide repeat units are dominant in *Brassica napus* [15] watermelon [16] and *Camellia sinensis* [17], whereas trinucleotide repeat units are dominant in the genomes of millet [18] and citrus [19]. At present, the main SSR repeats in the *G. sinensis* genome are dinucleotide repeats, which account for 56.25 and 60.86% of SSRs in male and female plants, correspondingly, followed by mononucleotide and trinucleotide repeat motifs. A previous study on the transcriptome SSR of *G. sinensis* demonstrated an SSR repeat element of the *G. sinensis* transcriptome, this SSR repeat element is mainly based on the dinucleotide repeats [20]. It has provided a new way for identifying the genetic relationship and phylogenetic evolution of the genus *Gleditsia*. Thus, the genomes and transcriptomes of *G. sinensis* are dominated by dinucleotide repeat units. The dominance of motifs in *G. sinensis* may be due to the motifs are rendered into certain amino acids in the process of translation, and the proteins that are present in these amino acids account for a large proportion of the species [16].

In recent years, research on *Gleditsia* has mainly focused on introduction and cultivation, reproductive technology, chemical composition analysis, stress resistance, and provenance test. The lack of molecular markers has limited the molecular identification and genetic diversity of *Gleditsia* germplasm resources and has affected the formulation of strategies for conserving *Gleditsia* germplasm resources. With the development of molecular biology, molecular markers are extensively investigated in various plant studies. The high polymorphism of SSR molecular markers combined with related biological analysis methods plays an important role in constructing a genetic map, genetic diversity determination, variety identification, seed purity detection, and marker-assisted breeding of medicinal plants. Lin et al. [20] constructed 18 DNA fingerprints of *G. sinensis* germplasm resources by using 8 polymorphic loci amplified by 3 primers, their results provided a theoretical basis for identifying the germplasm of *G. sinensis* and breeding new varieties [21]. However, to date, few reports on using microsatellite DNA sequence markers for early sex identification in plants are available. The application of DNA molecular marker technology in the sex identification of dioecious plants can improve the accuracy and reliability of the identification results, which are unaffected by development time and tissue specificity. The early sex identification of dioecious plants can obtain reliable identification information. Another study used a Microsatellite (GATA)_4_ probe to identify 1 band of 5 kb only in male papaya plants [22]. This previous study indicated that microsatellite markers can be used to identify male and female papaya. The accuracy rate of SSR markers for early sex identification of *Populus davidiana* is 100% [23]. Our results are consistent with the 100% accuracy of SSR markers associated with the sex chromosomes of *G. sinensis*. At present, by combining expressed sequence tags (EST) and SSR markers, the shortcomings of high cost in developing traditional SSR markers have been solved. The EST has a higher occurrence than the genomic SSR markers among species. In addition, a previous report indicated a primer EST-Eu059 related to the sex of a male *Eucommia ulmoides* plant, which was screened using 140 pairs of EST-SSR primers, thereby indicating that identifying the sex of plants is feasible using EST–SSR molecular markers [24].

Currently, little is known about the sex determination and differentiation mechanisms in the dioecious plant *G. sinensis*. Intensive studies of the regulation mechanisms of sex expression will provide important insights into the genetic regulation of *G. sinensis* and are useful for genetic studies and breeding applications. Particularly, the transcriptome sequencing strategy has been applied to detect differentially expressed genes of different sex types, such as those in Salix suchowensis [25], *Asparagus officinalis* [26], *Diospyros kaki* [27], and so on. We are now using this method to detect the sex-biased expression genes, and further analysis will be expected to add new insights of the genetic regulation of *G. sinensis* sex expression.

## Conclusions

The early morphological characteristics of *Gleditsia* plants are similar, thus limiting the potential use of these plants. In this study, SSR markers (i.e., 27, 34, 36, 39, and 41) related to the sex of a female *G. sinensis* plant have been screened out. These SSR markers may be used to detect the sex of a female plant in the early stage of development and have the advantages of 100% accuracy and efficiency. Simultaneously, the bands obtained through amplification demonstrate clear results, high repeatability, and robust stability. It provides accurate sex identification strategies between female and male plants, thus improving the utilization rate of *G. sinensis* resources.

## Material and methods

### Plant material

Complete and insect-free young leaves of *G. sinensis* (five males and five females) from the preserved forest at Xiaojing Town, Boai County, Henan Province, China, were collected to mark the sex and were brought to the laboratory in liquid nitrogen. In addition, 10 samples from Boai (five males and five males) and 20 samples from Xinxiang (10 males and females) were selected to validate the plant material and for further investigation. All land owners allow us to sample on their private land. The materials were identified as *G. sinensis* by Li at Henan Normal University, China. This material has been deposited in the biological specimen museum of Henan Normal University.

### DNA extraction

A Plant Genomic DNA Kit (Beijing BIOTEKE Biotechnology Corporation) was used to extract DNA from young leaves of *G. sinensis* according to the kit instructions. Take the leaf of *G. sinensis* in a mortar, add liquid nitrogen to fully grind it into a fine powder, transfer it to a centrifuge tube, add 550 μi Buffer P1 and 4 μL RNase A, mix vigorously for 1 min, and leave it at room temperature for 10 min. Add 130 μL of Buffer P2, mix vigorously for 1 min, and centrifuge at 12000 rpm for 3 min. Pipette the supernatant into separation column A, centrifuge at 12,000 rpm for 1 min, and collect the filtrate. Transfer the filtrate to a new centrifuge tube, add 1.5 volumes of Buffer P3 and mix well. Add the mixture to an adsorption column AC, centrifuge at 12,000 rpm for 1 min, and discard the waste in the collection tube. Add 500 μL of rinse solution WB, centrifuge at 12,000 rpm for 1 min, and discard the waste solution. Put the adsorption column AC back into the empty collection tube, centrifuge at 13,000 rpm for 3 min, remove the adsorption column AC, put it in a clean centrifuge tube, add 50 μL of elution buffer in the middle of the adsorption membrane, and leave it at room temperature for 3–5 min, Centrifuge at 12,000 rpm for 1 min to collect DNA. The purified DNA was quantified using a Nanodrop spectrophotometer.

Establishment of female and male DNA pools: According to the principle of BSA [28], an equal amount of DNA from the five female and male plants from Boai was mixed to form the female and male bulks, respectively. The two DNA bulks were fragmented by sonication. 400 bp fragments were recovered for library construction using Illumina Nextera library kit. Selective hybridization method (beads enrichment procedure) is used to enrich the SSR fragments in genomic libraries as described [29]. Eight probes, namely, P (AG)_10_, P (AC)_10_, P (AAC)_8_, P (ACG)_8_, P (AAG)_8_, P (AGG)_8_, and P (ACATAT)_6_, were used for SSR enrichment. The enriched genomic libraries were sequenced with pair-end 250 bp (PE250) using an Illumina MiSeq platform. Raw reads were filtered in accordance with the requirements.

### SSR search and statistics

A microsatellite identification tool (MISA) [30] was used to search for SSR loci from all sequences. The parameters used in the search were mono-10, di-6, tri-5, tetra-5, penta-5, and hexa-5. The maximum allowable interval between two SSRs in the composite sequence is set to 100 bp.

### SSR cluster analysis

We used Perl program to mask repetitive sequences and replaced them with the letter R. SSRs with a flanking sequence that is less than 20 bp were removed because short flanking sequences are unsuitable for alignment for similarity comparison. CDhit software was used to cluster the remaining sequences. Cluster analysis was performed with the sequence similarity of 95%, coverage of 70%, and vacancy penalty score of -gap 1-gep-ext 0. Among them, a sequence with two or more SSRs could be used to make separate statistical clustering groups.

### Identification of sex-linked SSRs

To identify sex-specific SSRs, the SSR-containing sequences of the male and female plants were independently clustered by CDhit software. The similarity of a nucleotide sequence is set to 80%, the coverage is 70%, and the vacancy penalty score is set to -gap 1-gep-ext 0. The clusters only detected in the female plants were considered female-specific SSRs, whereas the clusters only detected in the male trees were considered male-specific SSRs.

### SSR polymorphism assessment

Perl program was used to analyze the length of a repeat sequence in the clusters. If the lengths of repeat sequence in a cluster were the same, then the polymorphism of this cluster was scored as 1. If the lengths of the repeat sequence in a cluster were different, then the polymorphism of this cluster was scored as 2 if two lengths are available, 3 if three lengths are involved, and so on.

### SSR primer design

Primer 3 v2.3.6 was used to design primers [15] for SSR sequences in a cluster with a polymorphism score of ≥2. The PCR product was controlled in 100–400 bp, and the target fragment was amplified from the first base of the repeat sequence to the last five bases of the repeat sequence. Moreover, other parameters were defaults. All the primers were screened with the following criteria: (1) Single-nucleotide and complex repeat motifs are excluded from the primer design. (2) Each SSR marker only contains one SSR type to avoid the influence on polymorphism through the second SSR during amplification. (3) The designed primers must be within a cluster, and the length polymorphism of SSRs in clusters is more than 2. (4) Every designed primer must support two primers in clusters. (5) Identical primer results are removed.

### PCR amplification and agarose gel electrophoresis

A Kit (RR002A) from TAKARA was used for PCR amplification, PCR amplification was performed in a volume of 25 μL with 1.0 μL DNA template (40 ng·μl^− 1^), 15.5 μL ddH_2_O, 2.0 μL dNTP (0.00625 mM/μL), 1.0 μL forward primers (5 pmol), 1.0 μL reverse primers (5 pmol), 2.5 μL 10 × buffer, 1.5 μL MgCl2 (2.5 mM/mL), and 0.5 μL Taq DNA polymerase (5 U/μL). The PCR program is described as follows: denaturation at 95 °C for 5 min, then repeat with 30 cycles for 30 s at 95 °C, 52 °C, and 72 °C, with a final extension of 7 min at 72 °C. PCR products were electrophoretically separated on 1% agarose gels. Product sizes were estimated by comparison with a 700 bp DNA ladder.

## Data Availability

The datasets has been uploaded to the NCBI SRA database. SRA accession: PRJNA644493. SRA records will be accessible with the following link: https://www.ncbi.nlm.nih.gov/sra/PRJNA644493

## Abbreviations

SSR: Simple sequence repeat
BSA: Bulked segregant analysis
*G. sinensis*: *Gleditsia sinensis* Lam. (Leguminosae)
EST: Expressed sequence tags
